# Maintaining Integrity Under Stress: Envelope Stress Response Regulation of Pathogenesis in Gram-Negative Bacteria

**DOI:** 10.3389/fcimb.2019.00313

**Published:** 2019-09-04

**Authors:** Claire L. Hews, Timothy Cho, Gary Rowley, Tracy L. Raivio

**Affiliations:** ^1^School of Biological Sciences, University of East Anglia, Norwich, United Kingdom; ^2^Department of Biological Sciences, University of Alberta, Edmonton, AB, Canada

**Keywords:** Gram-negative bacteria, envelope stress, pathogenesis, sigmaE response, Cpx response

## Abstract

The Gram-negative bacterial envelope is an essential interface between the intracellular and harsh extracellular environment. Envelope stress responses (ESRs) are crucial to the maintenance of this barrier and function to detect and respond to perturbations in the envelope, caused by environmental stresses. Pathogenic bacteria are exposed to an array of challenging and stressful conditions during their lifecycle and, in particular, during infection of a host. As such, maintenance of envelope homeostasis is essential to their ability to successfully cause infection. This review will discuss our current understanding of the σ^E^- and Cpx-regulated ESRs, with a specific focus on their role in the virulence of a number of model pathogens.

## Introduction

Bacteria encounter numerous harsh and fluctuating environments during their lifecycle. This is of particular concern to pathogenic bacteria which must be able to tolerate the challenging conditions and immune response within a host. Detection of potentially harmful changes in the environment, or damage caused by such conditions, are inducers of bacterial stress responses. The ability of a bacterium to sense and withstand these environmental stresses is crucial to its survival, particularly within a host, and to establish and maintain infection. Within the host, a bacterium is threatened by a milieu of noxious stresses including temperature and pH changes, antimicrobial compounds, bile salts, oxidative and nitrosative stress, and nutrient starvation. In addition, successful colonization and infection of hosts requires careful regulation of a multitude of virulence factors, many of which are envelope-localized.

Consisting of an inner membrane (IM), periplasmic space (PP) containing a thin peptidoglycan (PG) layer and an outer membrane (OM), the Gram-negative bacterial envelope functions as the interface between the cell and the extracellular environment. It serves as the gate for import and export, barrier to harmful substances, protects against shifting environmental conditions and is the location of many essential metabolic processes. The envelope is first subjected to harsh environmental conditions and must be able to withstand the associated stresses. The ability to maintain envelope homeostasis and quickly repair any damage to ensure integrity is dependent on envelope stress responses (ESRs).

ESRs are able to perceive the presence of extracellular stress and the disruption of periplasmic homeostasis that can arise as a result (e.g., damage to proteins in the outer membrane or periplasm). The ESRs are comprised of a series of regulatory cascades, which have independent and overlapping stimuli, as well as regulon members. They are grouped into two component signal transduction (2CST) systems and RNA polymerase-associating alternative sigma factors. Much of our understanding of the mechanisms and regulons of the ESRs have arisen from extensive work in *Escherichia coli*. The best characterized ESRs are regulated through the σ^E^ pathway and the 2CST system CpxRA, central to the responses to outer membrane/periplasmic and inner membrane stress, respectively. In addition, other key ESRs include the regulator of capsule synthesis (Rcs) response, responding, in broad terms, to outer membrane and peptidoglycan stresses (Guo and Sun, 2017; Wall et al., 2018) and the phage shock protein (Psp) response, responding to disruptions at the IM (reviewed in Darwin, 2013; Flores-Kim and Darwin, 2016). Further 2CST systems exist, in addition to CpxAR, including ZraSR and BaeSR. Studies describe ZraSR as a zinc responsive system (Appia-Ayme et al., 2012; Petit-Härtlein et al., 2015), but this 2CST system has not been studied in depth and its precise physiological role remains to be determined. BaeSR on the other hand, responds to antibiotics/toxic compounds (Raffa and Raivio, 2002; Macritchie and Raivio, 2009; Appia-Ayme et al., 2011). Though not thought of as strictly responding to envelope stress, other regulatory systems such as the PhoPQ and EnvZ/OmpR two-component systems regulate processes that impact the ability of bacteria to adapt to envelope stress.

This review will summarize our current understanding of the σ^E^ and Cpx ESRs. While by no means the only ESRs relevant to bacterial pathogenesis, a large body of work has been conducted in characterizing the mechanism and function of these two ESRs in various Gram-negative bacteria. The aim of this review is to explain the basic mechanisms of these systems, as well as discuss their relevance to physiology and pathogenesis in several Gram-negative bacteria. In particular, we wish to highlight recent findings since this topic was last reviewed (Rowley et al., 2006; Raivio, 2014; Fang et al., 2016).

## Envelope biogenesis

Being the barrier between the intracellular and extracellular environment, proper formation and maintenance of the envelope is essential to bacterial viability during all stages of a pathogen's lifecycle. Envelope biogenesis is a complex process which involves several machineries (Silhavy et al., 2010). Here we briefly describe those that transport, fold and assemble envelope proteins, since misfolding and/or mis-localization of such proteins has been directly linked to induction of both the σ^E^ and Cpx ESRs.

Proteins destined for all compartments of the envelope begin their life in the cytoplasm. As such, these proteins must be transported into or across the IM. Two different pathways exist for protein transport across the IM; the Sec translocase pathway and the twin arginine translocase (Tat) pathway. The vast majority of secreted proteins utilize the Sec pathway (reviewed in Kuhn et al., 2017; Tsirigotaki et al., 2017), while the Tat system (reviewed in Lee et al., 2006; Palmer and Berks, 2012) transports proteins which have already been folded in the cytoplasm and those which also contain metal cofactors. Briefly, an unfolded protein is targeted to the Sec translocase machinery through the recognition of an N-terminal signal peptide. SecYEG forms the IM channel through which unfolded proteins are transported. Transport across the IM is energized by the ATPase SecA. Proteins that are inserted into the IM can be inserted by SecYEG itself or utilize the IM protein YidC to assist in insertion. Once secreted across the IM, proteins can be modified and translocated in several different ways. A number of factors including the IM protein insertase YidC, together with proteases and associated regulators, play roles in the biogenesis and quality control of IM proteins (Akiyama, 2009; Luirink et al., 2012).

Lipoproteins are a special class of envelope proteins that are acylated at their N-terminus and can be localized to either the IM or the OM (reviewed in Okuda and Tokuda, 2011; Szewczyk and Collet, 2016; Narita and Tokuda, 2017). In brief, after being secreted across the IM, lipoproteins undergo multiple modification steps that result in the addition of several acyl chains to an N-terminal cysteine residue. OM-destined lipoproteins are trafficked to the OM by the Lol pathway. An IM complex consisting of the ATP-binding cassette (ABC) transporter LolCDE recognizes OM-destined lipoproteins based on the presence of an aspartate residue at the +2 residue and energizes the transfer of these proteins to a periplasmic chaperone LolA. LolA transports OM lipoproteins to the OM, where LolB, itself an OM lipoprotein, receives and inserts OM lipoproteins into the OM. As OM lipoproteins have been implicated in promoting the virulence of some Gram-negative organisms, the Lol pathway presents a potential target for the development of new therapies. For example, the surface-exposed lipoproteins of *Neisseria* spp. play roles in pathogenesis, such as immune evasion (reviewed in Hooda et al., 2017). More broadly, lipoproteins make up key members of envelope biogenesis machineries, such as the BAM complex (see below), and can indirectly impact many envelope-localized virulence determinants.

Outer membrane biogenesis has been reviewed extensively elsewhere (Ruiz et al., 2006; Bos et al., 2007; Plummer and Fleming, 2016; Konovalova et al., 2017) therefore it will only be briefly described in this review for relevance to understand the contribution of the ESR to this process. All unfolded outer membrane proteins (uOMPs) are translocated via the Sec system. Once the uOMP reaches the periplasm, the signal sequence is cleaved and the nascent OMP is delivered by periplasmic chaperones (SurA and Skp) to the β-barrel assembly machinery (BAM) complex for folding and insertion into the outer membrane.

The BAM complex consists of five major components: BamA (YaeT), BamB (YfgL), BamC (NlpB), BamD (YfiO), and BamE (SmpA) (Plummer and Fleming, 2016). The genes encoding the BAM complex components are all regulated by σ^E^ (Skovierova et al., 2006; Lewis et al., 2008) and are discussed below. BamA, a member of the Omp85 protein family and originally identified in *Neisseria meningitidis*, is highly conserved across Gram-negative bacteria and is essential to the complex and bacterial viability (Voulhoux et al., 2003). Interestingly, while BamA is essential across all Gram-negative bacteria, the essentiality of the other four lipoprotein components varies; for example, BamD is essential for complex function in *E. coli* yet is not essential in *Salmonella* (Fardini et al., 2009). BamA formation is dependent on BamD with depletions in this lipoprotein resulting in BamA misfolding (Misra et al., 2015). In addition, BamB and BamD have been shown to bind unfolded BamA and assist in its localization and insertion into the outer membrane (Hagan et al., 2013).

Aside from its essentiality, there are numerous links between the BAM complex and pathogenicity. *Salmonella bamE* mutants are attenuated in mice (Lewis et al., 2008) and *Salmonella* Enteritidis *bamB* and *bamD* deletions render the pathogen less virulent with reduced expression of flagella and the type III secretion system (T3SS) (Amy et al., 2004; Fardini et al., 2007, 2009). In *Yersinia enterocolitica*, a *bamB* mutant is attenuated in mice with a significant reduction in the spleen bacterial burden after 3 days (Weirich et al., 2017). Additionally, a *bamE* deletion leads to OMP defects and rifampicin sensitivity in *E. coli* (Sklar et al., 2007a).

Recently, a number of studies have identified small molecule inhibitors of components of the BAM complex. Hagan et al. (2015) identified a 15-mer peptide fragment of BamA that binds to BamD. Since BamA formation is dependent on BamD and BamA is required for cell viability, substrate competition between the small molecule and the BamA substrate resulted in growth defects and increased sensitivity to vancomycin and rifampicin. BamA has been identified as a viable target for therapeutics with the development of a monoclonal antibody (MAB1) which binds to the extracellular loop of BamA and affects OM integrity (Storek et al., 2018). Furthermore, inhibition of *Pseudomonas* BamA by bacteriocins has also been observed, specifically lectin like bacteriocins (LlpAs). Bacteria often secrete these toxic compounds in order to selectively kill related bacteria and Ghequire et al. (2018) identified that LlpA resistant *Pseudomonas* carried mutations in the extracellular loop of BamA.

Put together, studies such as these support the consensus that understanding the process of OM biogenesis and its maintenance may provide new therapeutic routes against these pathogens.

## The Extracytoplasmic Sigma Factor σ^E^

**Figure 1:**
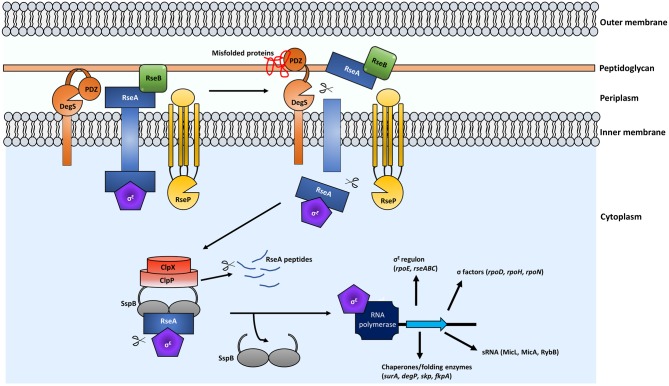
Regulated intramembrane proteolysis (RIP) leads to activation of the σ^E^ ESR. In the presence of misfolded proteins, a conformational change as a result of misfolded proteins binding to the PDZ domain of DegS occurs. Subsequently the protease domain of DegS is exposed and the periplasmic domain of RseA is cleaved. RseP then cleaves RseA at the cytoplasmic domain, releasing the σ^E^-bound RseA portion into the cytoplasm. Binding of the adaptor protein SspB to RseA-σ^E^ recruits the ClpXP protease for degradation of RseA and release of free σ^E^. σ^E^ binds to RNA polymerase and transcription of the σ^E^ regulon is induced.

Maintenance of the outer membrane depends largely on the extracytoplasmic sigma factor σ^E^ (*rpoE*) (Rouvière et al., 1995). This ESR senses misfolded OMPs within the outer membrane and periplasmic space. Activation of the σ^E^ pathway involves a series of proteolytic cleavage events (Figure 1). Inducers of the σ^E^ pathway are summarized in Rowley et al. (2006) and include: oxidative stress, heat shock, carbon starvation and biofilm formation. More recently, acid stress (Muller et al., 2009) ultraviolet A (UVA) radiation, P22 phage and hypo-osmotic shock have also been shown to induce the σ^E^ ESR (Amar et al., 2018). Generally, activation of the σ^E^ pathway is due to accumulation of misfolded and/or mis-translocated OMPs or LPS within the periplasm (Rowley et al., 2006). In laboratory *E. coli* strains and *Yersinia enterocolitica, rpoE* is an essential gene (De Las Peñas et al., 1997a; Heusipp et al., 2003), although suppressor mutations do enable *rpoE* mutants to be viable (De Las Peñas et al., 1997a). Insertion mutations in *ydcQ*, a putative DNA binding protein (Button et al., 2007), and two genes of unknown function *yhbW* and *ptsN* (Hayden and Ades, 2008) have been identified to enable the *rpoE* deletion in *E. coli* to be tolerated. Numerous studies have shown that *rpoE* is not essential in other bacteria including *Salmonella* (Humphreys et al., 1999; Skovierova et al., 2006), although it does make a major contribution to their virulence. Interestingly, it has recently been reported that loss of the LPS O-antigen renders an *rpoE* deletion lethal in *Salmonella* (Amar et al., 2018). The authors propose that when present, the LPS O-antigen provides protection to the *Salmonella* OM allowing an *rpoE* deletion, and presumably the OM defects that result, to be tolerated.

Encoded within the *rpoE* operon are the σ^E^ inhibitors *rseA* and *rseB* (Dartigalongue et al., 2001). Activation of the σ^E^ ESR occurs via regulated intramembrane proteolysis (RIP) and begins at the transmembrane protein RseA (Figure 1). Under normal physiological conditions, RseA is bound to σ^E^ and as such, sequesters it from interacting with RNA polymerase to influence gene expression. RseB is also a negative regulator of the σ^E^ pathway and when bound to RseA it increases affinity of this complex for σ^E^, resulting in inhibition of the pathway (De Las Peñas et al., 1997b; Collinet et al., 2000; Ahuja et al., 2009; Chaba et al., 2011) (Figure 1).

In the presence of misfolded proteins, the σ^E^ cascade begins with the cytoplasmic cleavage of RseA by the protease DegS (Ades et al., 1999; Alba et al., 2002; Kanehara et al., 2002; Li et al., 2009). The DegS site-1 protease (S1P) is a transmembrane protein embedded within the IM (Figure 1). The presence of uOMPs in the periplasm induces conformational changes in DegS, via interaction between the C-terminus of the uOMPs with the PDZ protease domain of DegS. This conformational change exposes the protease region for RseA cleavage (Walsh et al., 2003; Sohn et al., 2009). DegS is essential in *E. coli* laboratory strains however construction of a *degS* mutant has been reported in certain extraintestinal *E. coli* strains, *Salmonella* Typhimurium (Rowley et al., 2005), and *Vibrio cholerae* (Mathur et al., 2007). Although viable, *S*. Typhimurium *degS* mutants are defective in their ability to colonize the host and cause infection (Redford et al., 2003) while *V. cholerae degS* mutants are unable to activate the σ^E^ cascade following antimicrobial peptide (AMP) treatment (Mathur et al., 2007). Interestingly, *degS* mutants can still mount a σ^E^ response to certain stresses (Rowley et al., 2005). In *S*. Typhimurium, σ^E^ can be activated by acid stress independently of DegS and misfolded proteins (Muller et al., 2009).

**Figure 2:**
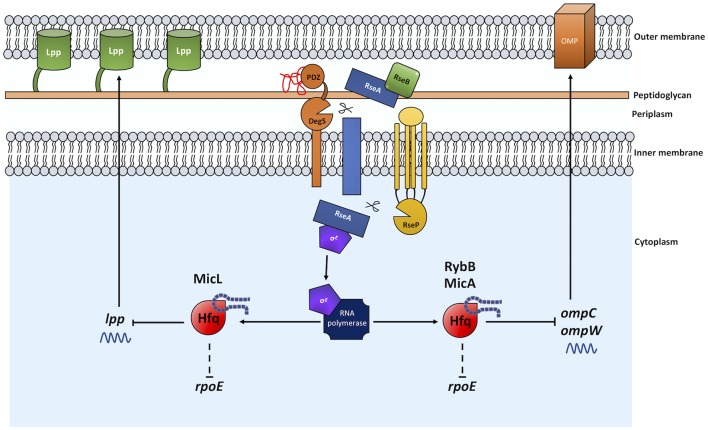
The sRNAs MicL, RybB, and MicA exert control over OM homeostasis and the σ^E^ envelope stress response. The presence of misfolded OMPs activates the σ^E^ ESR through a proteolytic cleavage cascade as described in Figure 1. The σ^E^ regulon includes the Hfq-associating sRNAs *micL, rybB, and micA*. MicL inhibits *lpp* mRNA translation leading to a reduction in Lpp production. RybB and MicA reduce *ompC* and *ompW* mRNA levels, reducing the flux of uOMPs into the periplasm during stress and σ^E^ inducing conditions. Normal arrows represent positive regulation and blunt arrows indicate negative regulation. Dashed lines indicate where regulation is indirect and/or where intermediate factors are unknown.

Following cleavage by DegS, the RseP (YaeL) protease is recruited and the cytoplasmic portion of RseA, bound to σ^E^, is released into the cytoplasm (Alba et al., 2002; Kanehara et al., 2002) (Figure 1). RseP is a site-2 protease (S2P), therefore, its cleavage of RseA can only occur after cleavage by the S1P. Li et al. (2009) identified that S1P cleavage leads to S2P cleavage due to the exposure of a hydrophobic amino acid (Val-148) at the carboxyl-terminus of RseA. Inhibition of RseP prevents cleavage of RseA and results in a lethal sequestration of σ^E^. Interestingly, a sRNA, RseX, was shown to reduce the levels of OMPs OmpA and OmpC when overexpressed and this led to survival of *rseP* mutant cells in *E. coli* (Douchin et al., 2006). Recently, Konovalova et al. (2018) identified a eukaryotic matrix metalloprotease (MMP) inhibitor which targets RseP and leads to toxic uOMP accumulation. This was found to be due to loss of σ^E^-regulated sRNAs, MicA and RybB, which play an important role in downregulating expression of uOMPs (see Figure 2 and described in detail below).

Following release of RseA bound σ^E^, the adaptor SspB binds to the complex and recruits the ATP-dependent ClpXP protease (Figure 1) (Flynn et al., 2004; Baker and Sauer, 2012). ClpX is an AAA+ ATPase and ClpP is a peptidase (Baker and Sauer, 2012) and in the final stage of the σ^E^ cascade the protein complex releases σ^E^ from RseA (Chaba et al., 2007). Analysis of the roles of the ClpXP protease provides evidence that individual components of the σ^E^ pathway play important roles in bacterial pathogenesis with *Salmonella clpX* and *clpP* mutants being attenuated in macrophages and BALB/c mice (Yamamoto et al., 2001).

Once released from RseA, free σ^E^ binds RNA polymerase as a cofactor, subsequently inducing expression of the σ^E^ regulon (Figure 1). The σ^E^ regulon has been linked with virulence in a number of pathogens (discussed below). The function of proteins that form part of the σ^E^ regulon across many bacterial species include those for DNA repair, metabolism, OM biogenesis and chaperones and proteases required for maintaining periplasmic homeostasis (Dartigalongue et al., 2001; Rhodius et al., 2006; Skovierova et al., 2006).

σ^E^-regulated chaperones and proteases are of importance to OM biogenesis and the tolerance of extracytoplasmic stress particularly during host infection (see below). The major σ^E^-regulated chaperones are Skp, SurA, FkpA, and HtrA (DegP). The function of these proteins has been studied extensively in *E. coli*, however they are well-conserved across multiple Gram-negative species including *E. coli, Salmonella*, and *Yersinia* sp.

SurA possesses peptidyl-prolyl cis/trans isomerase (PPIase) activity which folds proteins via catalysis of the rate-limiting cis/trans isomerization of peptidyl bonds around proline residues (bacterial PPIases are reviewed in Ünal and Steinert, 2014). In addition to, and independently of, its PPIase activity SurA also functions as a periplasmic chaperone (Behrens et al., 2001). A major role of the periplasmic chaperones is to prevent aggregation of uOMPs; however, SurA and a further chaperone Skp, encoded within the *bamA* genomic region, have been shown to play a role in uOMP folding in cooperation with the BAM complex. Studies in *E. coli* have shown that SurA interacts with BamA in order to deliver uOMPs (Sklar et al., 2007b; Vuong et al., 2008), while Skp assists in the folding of OmpA and Skp-OmpA complexes interact with BamA (Patel and Kleinschmidt, 2013). Interestingly, double deletion mutants of *skp* and *surA* result in a lethal phenotype and as a result, it is understood that they function in two different but overlapping pathways of OM biogenesis (Sklar et al., 2007b). Despite this, overexpression of *fkpA* can compensate for the lethal phenotype and enables *Salmonella* growth at 37°C (Ge et al., 2014). Like SurA, FkpA is a PPIase and chaperone and its chaperone activity is independent of PPIase activity. In addition to a role in OM biogenesis, overexpression of FkpA was shown to rescue *degP* (*htrA*) mutants during heat shock (Arie et al., 2001). DegP is unique in its ability to function as both a chaperone and a protease and this switch is dependent on temperature. At low temperatures, DegP is a chaperone transporting misfolded and unfolded OMPs; however, at higher temperatures it possesses protease function degrading proteins that are beyond repair (Spiess et al., 1999).

In addition to chaperones and proteases, encoded within the *rpoE* regulon are a number of sRNAs with regulatory functions. sRNAs bind to specific mRNA targets and subsequently activate or repress the mRNA through affecting its stability or by inhibiting its translation (Holmqvist and Wagner, 2017). sRNAs have been shown to regulate the σ^E^, CpxRA, and Rcs ESRs (reviewed in Frohlich and Gottesman, 2018). The sRNAs regulated by σ^E^ are MicA (SraD), RybB and MicL (RyeF/SlrA) and they function to regulate the σ^E^ response and expression of a group of OMPs and lipoproteins (Figure 2). Ultimately, the sRNAs regulated by σ^E^ downregulate other processes, such as uOMP production, that may otherwise lead to continued activation of the ESR. All σ^E^-regulated sRNAs associate with the RNA chaperone Hfq and inactivation of Hfq is an activator of the σ^E^ ESR (Figueroa-Bossi et al., 2006; Sittka et al., 2007; Klein and Raina, 2015). This Hfq-dependent regulation does indeed warrant further study in order to better understand the underlying mechanisms. In *Salmonella*, loss of Hfq has been shown to result in an increase in DegS-dependent cleavage of RseA, and this is likely due to increased accumulation of uOMPs (Figueroa-Bossi et al., 2006). In addition, Guisbert et al. (2007) identified that downregulation of the mRNA of eight OMPs (*tsx, fiu, ompX, ompA, ompF, lpp, ompC, yhcN*) is dependent on Hfq. Therefore, through association with sRNAs that function to downregulate uOMP production (described in more detail below) and control the σ^E^ response, Hfq can indirectly inactivate σ^E^.

MicL is an 80 nt transcript, processed from a primary transcript of 308 nt, and is located within the *cutC* gene. Also referred to as SlrA (suppressing lap RNA), MicL can function as a multicopy suppressor of *lapA lapB* mutations (the Lap proteins are essential in the process of LPS synthesis and for cell viability) (Klein et al., 2014). To date, the only known mRNA target of MicL is *lpp* (Guo et al., 2014). The *lpp* gene encodes an OM lipoprotein, commonly referred to as Lpp or Braun's lipoprotein (Braun, 1975), which is covalently attached to the peptidoglycan layer. Lpp is the most abundant protein in *E. coli* and functions to stabilize the cell envelope through its OM-peptidoglycan interaction (for recent review, see Asmar and Collet, 2018). MicL targets *lpp* by preventing translation of its mRNA, thereby inhibiting Lpp protein production (Guo et al., 2014). This could be seen as counterproductive; why would σ^E^, a mechanism in place to maintain OM integrity, prevent production of a cell envelope stabilizing protein? Studies indicate that in doing so, demand on the Lol lipoprotein assembly machinery is lessened. This, in turn, enables increased production of the BamD lipoprotein and LPS assembly components (primarily LptE), thus increasing the folding of uOMPs and LPS production in the periplasm, which would otherwise further induce σ^E^ (Guo et al., 2014).

The remaining sRNAs MicA and RybB were first identified in *E. coli* as ~70 nt and ~80 nt, respectively. Since, these sRNAs have also been identified and shown to be conserved in *Salmonella* (Papenfort et al., 2006). These sRNAs overlap in function and downregulate expression of outer membrane porins in an Hfq-dependent manner. MicA is responsible for the decrease in *ompA* mRNA, particularly during stationary phase when σ^E^ is highly active (Vogel and Papenfort, 2006). RybB has been shown to specifically decrease *ompC* and *ompW* mRNA levels via Hfq (Johansen et al., 2006). As a result, these sRNAs contribute to the maintenance of envelope homeostasis via downregulation of OMP production, in a similar fashion to MicL, reducing transport of uOMPs into the periplasm during conditions of stress when the periplasmic uOMP content may already be elevated.

Interestingly, MicA negatively regulates the response regulator PhoP of the PhoPQ 2CST via base-pairing within the *phoP* translation initiation site (Coornaert et al., 2010). The contribution of the PhoPQ 2CST system to OM remodeling in *Salmonella* has been well-documented (Ernst et al., 2001; Dalebroux and Miller, 2014; Dalebroux et al., 2014) and cross-talk between this system and the σ^E^ ESR provides further evidence that different ESR pathways overlap. This connection emphasizes the necessity of a coordination of responses that modify and maintain the envelope during infection and bacterial stress. The negative regulation of the PhoPQ 2CST system by σ^E^ demonstrates that under some ESR-inducing conditions, it may be detrimental to the bacterium for certain ESRs to be simultaneously active.

### σ^E^ and Bacterial Pathogenesis

#### Salmonella

*Salmonella* is an intracellular pathogen capable of causing infection in both humans and animals. Serovars of *Salmonella* sp. can reside in different niches and vary in the type of infection they cause. *S*. Typhimurium and *S*. Enteritidis are both examples of enteric bacteria typically causing food poisoning, while *S*. Typhi is responsible for the more serious and systemic Typhoid fever.

**Figure 3:**
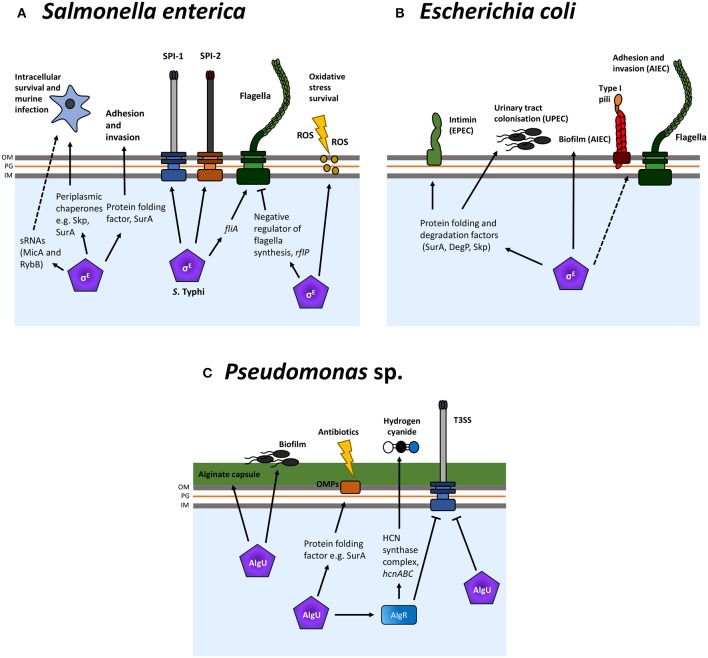
σ^E^-regulation of virulence factors in *Escherichia coli, Salmonella enterica*, and *Pseudomonas* spp. The σ^E^ ESR regulates many virulence factors and surface structures, such as adhesins, in multiple bacteria such as *Salmonella enterica* **(A)**, *E. coli* **(B)**, and *Pseudomonas* spp. **(C)**, primarily via the upregulation of periplasmic folding factors. Additionally, a number of bacteria-specific factors are regulated by this ESR. These include hydrogen cyanide in *Pseudomonas* sp. and the *Salmonella* pathogenicity islands (SPIs), SPI-1, and SPI-2. Normal arrows represent positive regulation and blunt arrows indicate negative regulation. Dashed lines indicate where regulation is indirect and/or where intermediate factors are unknown.

A large number of σ^E^-regulated proteins, chaperones and PPIases have been implicated in *Salmonella* virulence (Figure 3A). During infection, *Salmonella* resides within macrophages in the *Salmonella* containing vacuole (SCV), a site of ROS production and associated stress. It has been shown previously that σ^E^ is important for survival within macrophages and in a murine infection model (Humphreys et al., 1999). *rpoE* is upregulated in macrophages (Eriksson et al., 2003) and the σ^E^ regulon is required for *Salmonella* resistance to oxidative stress (Testerman et al., 2002; Li et al., 2015a). Furthermore, in the invasive serovar *S*. Typhi, responsible for the systemic infection Typhoid fever, *rpoE* mutants are attenuated for invasion and intracellular survival. In addition, expression of the pathogenicity islands SPI-1 and SPI-2, encoding the T3SSs required for invasion and intracellular survival, are reduced (Xie et al., 2016; Zhang et al., 2016). σ^E^ also plays an important role in the downregulation of cellular processes. The anti-FlhC_2_D_4_ complex factor RflP (YdiV) is activated by σ^E^ and RflP functions to target the FlhC_2_D_4_ master regulator of flagellar synthesis to the ClpXP protease for degradation in *S*. Typhimurium (Spöring et al., 2018). As such, in this non-typhoidal serovar, σ^E^ is involved in the downregulation of *Salmonella* motility. Ultimately, research suggests that downregulation of flagellar synthesis can aid in host immune evasion, thus increasing bacterial fitness during infection. Conversely, in *S*. Typhi, σ^E^ has been found to promote flagellar gene expression, during osmotic stress, via upregulation of *fliA* (Du et al., 2011). The authors of this study propose that RpoE may respond to the hyperosmotic environment in the intestinal lumen and increase motility to enable *S*. Typhi invasion of epithelial M cells. These interesting discrepancies indicate that σ^E^ may be involved in differences between localized and systemic *Salmonella* infections. Much of the groundwork in understanding the role of σ^E^ in *Salmonella* pathogenesis has been performed in *S*. Typhimurium; however, these data indicate the continuing need to expand these studies into other serovars, especially those which cause invasive and systemic disease.

Further links between σ^E^ and coordination of virulence gene expression have also been shown: the SPI-2 pathogenicity island is, in part, regulated by the SsrAB 2CST system and an *S*. Typhimurium *rpoE* mutant has reduced expression of the SsrB-dependent secreted effector protein SseB (Osborne and Coombes, 2009). As described above, encoded within the *bamA* region is the σ^E^-regulated periplasmic chaperone, *skp*. Rowley et al. (2011) identified significant attenuation of a *skp* mutant in a murine infection model. Additionally, *fkpA* mutants of *Salmonella* Copenhagen show reduced levels of intracellular survival (Horne et al., 1997), although studies in *S*. Typhimurium demonstrate that *fkpA* mutants are unaffected for intracellular survival, unless also combined with a deletion in *surA* or *degP* (Humphreys et al., 2003). Deletion of *surA* leads to loss of adhesion and invasion of epithelial cells by *S*. Typhimurium (Sydenham et al., 2000). In addition, the *surA* mutant was identified as an attenuated live vaccine. Sydenham et al. (2000) showed that when mice were challenged with WT *S*. Typhimurium, following a challenge with the *surA* mutant, the WT was unable to colonize the host. These findings certainly point toward an important role for σ^E^-regulated periplasmic chaperones in infection and also for the identification of new therapeutic targets.

In addition to the proteins regulated by σ^E^, the sRNAs have also been linked to virulence. MicA and RybB are both upregulated inside macrophages (Srikumar et al., 2015). Furthermore, MicA has a role in biofilm formation in *Salmonella* and this was understood to be due to MicA targeting of *ompA* and *phoP* mRNAs (Kint et al., 2010).

Aside from a role in pathogenicity and intracellular survival, *rpoE* is also required for *Salmonella* resistance to antimicrobial peptides (AMPs) during infection. Specifically, *rpoE* mutants were shown to be reduced in survival when challenged with the bactericidal/permeability-increasing protein (BPI)-derived peptide P2 and the murine α-defensin cryptdin-4 (Crp4), both of which are known to disrupt the cell envelope (Crouch et al., 2005).

Studies identifying the role of previously uncharacterized σ^E^-regulated genes are continually emerging. Recently, Morris et al. (2018) demonstrated that the σ^E^-regulated lipoprotein YraP contributes to OM integrity and the ability to cause murine infection. Results showed that infection of mice with a *yraP* mutant resulted in significant attenuation, particularly in the liver, compared to WT. However, the *yraP* mutant strain was not attenuated for adhesion, invasion or intracellular survival in J774, Caco-2 or primary bone marrow-derived macrophages. As such, the precise mechanism behind the attenuation of Δ*yraP* in mice is yet to be elucidated.

#### Escherichia coli

The role of σ^E^ in the pathogenesis of *E. coli* has not been studied to the same extent as in *Salmonella*, potentially because *rpoE* is an essential gene in laboratory *E. coli*. However, a small number of studies have demonstrated a role for σ^E^-regulated chaperones in the virulence of different pathogenic *E. coli* strains (Figure 3B).

Extraintestinal *E. coli* strains are capable of colonizing and causing infection outside of their usual intestinal niche. Uropathogenic *E. coli* (UPEC) is one such example and is a major cause of urinary tract infections (UTIs). It has been described that the σ^E^-regulated chaperones *degP* and *skp* are important for *E. coli* urinary tract colonization (Redford and Welch, 2006). Enteropathogenic *E. coli* (EPEC) is a major cause of infant diarrhea and its ability to adhere to intestinal enterocytes is central to its pathogenesis (Walsham et al., 2016). The adhesin intimin is required for attachment to epithelial cells and it has been shown that the σ^E^-regulated chaperones SurA, DegP and Skp are needed for the insertion of intimin into the OM (Bodelon et al., 2009).

Another *E. coli* pathovar, adherent-invasive *Escherichia coli* (AIEC), is capable of invading intestinal epithelial cells (Yang et al., 2017). σ^E^ is required for biofilm formation of Crohn's disease associated AIEC; inhibition of σ^E^ by overexpression of RseAB resulted in a significant reduction of AIEC biofilm formation (Chassaing and Darfeuille-Michaud, 2013). In addition, σ^E^ is important for AIEC adhesion and invasion of epithelial cells (Rolhion et al., 2007; Chassaing and Darfeuille-Michaud, 2013). Studies demonstrate that inhibition of σ^E^ leads to a reduction in adhesion and invasion and that this is in part, due to reduced expression of flagella and type 1 pili. However, the exact mechanisms behind the observed phenotypes are yet to be fully elucidated (Chassaing and Darfeuille-Michaud, 2013). In contrast to σ^E^, other stress responses including the 2CST system CpxAR have been implicated in *E. coli* virulence to a greater extent and this will be described in detail below.

#### Pseudomonas

*Pseudomonas aeruginosa* is an opportunistic pathogen associated with high morbidity and mortality in patients with underlying respiratory disease such as cystic fibrosis (Gellatly and Hancock, 2013). *P. aeruginosa* strains exist in mucoid or non-mucoid forms but generally environmental strains and those which lead to initial colonization of the host are non-mucoid in nature (Rao et al., 2011). It has been demonstrated that the conversion to the mucoid form occurs during host colonization and it appears that the conditions, particularly in the CF lung, positively select for mucoid *P. aeruginosa*. As expected, mucoid strains are most commonly associated with CF patients; however, they have also been isolated from non-CF patients (Govan and Deretic, 1996).

The conversion from non-mucoid to mucoid is mediated by mutations in *mucA*. The *mucA* gene forms part of the *algU, mucA, mucB, mucC* operon which is homologous to the *rpoE rseA rseB rseC* operon in *E. coli* (Rowley et al., 2006). Normally, MucA sequesters AlgU activity (as does RseA for RpoE); however, mutations in *mucA*, typically *mucA22*, result in constitutive *algU* activation (Mathee et al., 1999). The *algU* regulator, sharing 79% amino acid sequence homology with *E. coli* σ^E^ (Potvin et al., 2008), controls expression of the alginate biosynthesis operon, formed of 12 genes for biosynthesis and export which starts at *algD*. Induction of the AlgU pathway results in production of the exopolysaccharide alginate and is of particular importance for bacterial protection and persistence in the lung (Figure 3C).

Activation of the AlgU pathway occurs via RIP (reviewed in Damron and Goldberg, 2012), as does activation of the σ^E^ pathway. In addition, *Pseudomonas* has functional equivalents of the *E. coli* proteases DegP, DegS and RseP; MucD, AlgW, and MucP, respectively (Pandey et al., 2016). Further to this, the ClpXP proteases are conserved in *Pseudomonas* and are required to release AlgU from MucA in the cytoplasm (Qiu et al., 2008).

Yu et al. (1995) showed that *Pseudomonas algU* mutants can be complemented with *E. coli rpoE*, and this complementation provided the *algU* mutant with resistance to paraquat-induced ROS stress. It has not yet been shown whether the lethal *rpoE* deletion in *E. coli* can be rescued by *algU*.

Environmental stress has been shown to induce mutations in *mucA*. Mathee et al. (1999) described that when a non-mucoid strain, PA01, was grown in a biofilm treated with hydrogen peroxide, mucoid variants with mutations in *mucA* formed. The authors propose that these findings suggest anti-oxidant therapy may be a therapeutic option for patients, as this may aid in preventing the switch from non-mucoid to mucoid.

In addition to regulating alginate production, AlgU regulates genes required for virulence (Figure 3C). Interestingly, an *algU* deletion increases systemic virulence (Yu et al., 1996), hence it appears that *algU* is required for infection in the respiratory but not systemic environment. The ability to form a biofilm is a major virulence factor of many bacterial pathogens and AlgU is important for biofilm formation, specifically in non-mucoid strains (Bazire et al., 2010). AlgR is a component of the 2CST system AlgZR and is regulated by AlgU (Okkotsu et al., 2014). AlgR, in turn, regulates hydrogen cyanide (HCN) production which studies indicate is an important *Pseudomonas* virulence factor. Produced under low oxygen conditions, HCN has been shown to be responsible for killing in the *Caenorhabditis elegans* infection model (Gallagher and Manoil, 2001). Additionally, both AlgR and AlgU have been shown to suppresses the *Pseudomonas* T3SS in mucoid strains (Okkotsu et al., 2014).

Several of the chaperones and proteases involved in maintenance of OM and periplasmic homeostasis in *E. coli* and *Salmonella* are conserved in *Pseudomonas* sp. A recent paper described a *surA* deletion re-sensitizes a MDR strain to antibiotics, suggesting that SurA could be a promising therapeutic target (Klein et al., 2019). Moreover, in support of this conclusion, deletion of *surA* also increased sensitivity to normal human serum.

#### Conclusions and Further Evidence of Roles of σ^E^ in Bacterial Virulence

The σ^E^ ESR has been implicated in other bacterial species in addition to those described above. Unfortunately, due to limitations in space, a detailed discussion is beyond the scope of this particular review. In brief, additional examples of a role for σ^E^ in pathogenesis include work in *Vibrio cholerae* and *Yersinia* sp. In *Vibrio cholerae*, an *rpoE* deletion resulted in a highly attenuated strain that was unable to colonize the intestine (Kovacikova and Skorupski, 2002). Moreover, a σ^E^-regulated sRNA, VrrA, inhibits production of OmpA and subsequently inhibits outer membrane vesicle (OMV) formation (Song and Wai, 2009). VrrA was also shown to control biofilm formation through translational repression of *rbmC*, a *V. cholerae* specific biofilm matrix protein. When VrrA was overexpressed, biofilm levels were reduced and, as such, VrrA may assist in the transition of *V. cholerae* from attachment in the intestine to shedding and uptake by new hosts (Song et al., 2014). In *Yersinia* sp. the σ^E^-regulated chaperone SurA is required for *Y. pseudotuberculosis* adhesion to HeLa cells (Obi and Francis, 2013) and a *Y. pestis surA* mutant is attenuated in mice (Southern et al., 2016). The vast array of studies highlighted in this review demonstrate that σ^E^ is of extreme importance to not only survival of Gram-negative bacteria, through the maintenance of the extracytoplasmic OM, but also to the ability to cause such successful and varied infections.

## The CpxRA two-component system

**Figure 4:**
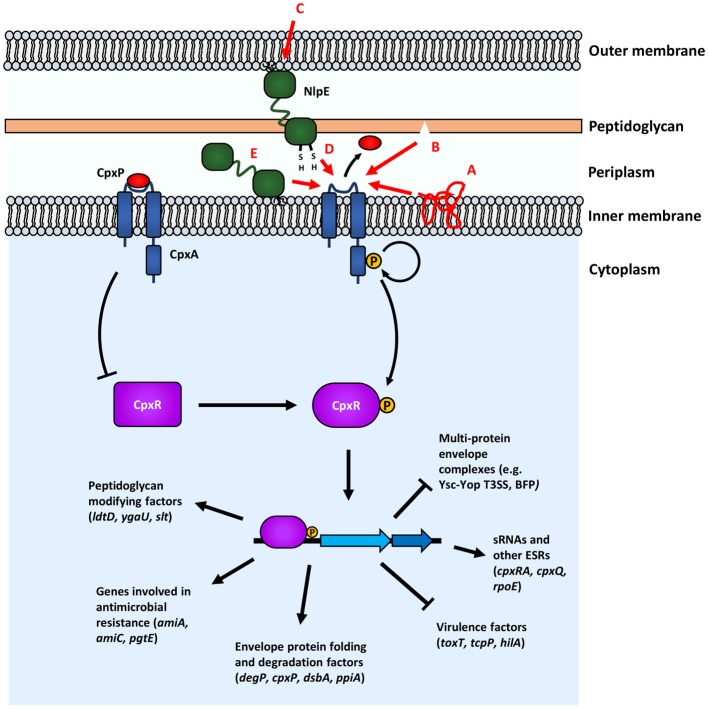
The Cpx response senses and responds to a variety of envelope stresses. Proposed inducing cues of the Cpx response are shown with red arrows and corresponding red lettering: (A) stresses that lead to protein misfolding; (B) damage to peptidoglycan; (C) surface signals; (D) monitoring periplasmic redox states; (E) OM lipoprotein trafficking defects. In the absence of induction, the phosphatase activity of CpxA keeps CpxR unphphosphorylated. Upon induction, phosphotransfer from phosphorylated CpxA to CpxR leads to CpxR-P modulation of transcription of genes involved in several processes. Genes shown are representative, but not exhaustive of the Cpx regulon.

The Cpx (conjugative pilus expression) response is a widely-conserved ESR in Gram-negative bacteria. As a canonical 2CST system, signaling in the Cpx response occurs through the sensor histidine kinase (SHK) CpxA and the response regulator CpxR (Figure 4). Under non-inducing conditions, the phosphatase activity of CpxA keeps CpxR in an unphosphorylated state (Raivio and Silhavy, 1997). Under inducing conditions, CpxA autophosphorylates and transfers the phosphate group to CpxR. One of the earliest findings implicating the Cpx response in a concrete role in responding to envelope stress was the finding that activated alleles of *cpxA* suppress the toxicity of the IM-localized fusion protein LamB-LacZ-PhoA (Cosma et al., 1995). Since then, much work has been done to characterize the function of the Cpx response in mitigating envelope stress (which is more comprehensively reviewed in Vogt and Raivio, 2012; Raivio, 2014). A variety of inducing cues activate the Cpx response, many, although not all, of which are thought to affect the integrity of the envelope and/or protein-folding outside of the cytoplasm. These include alkaline pH (Danese and Silhavy, 1998), aberrant expression of the Pap pilus (Jones et al., 1997), adhesion to hydrophobic surfaces (Otto and Silhavy, 2002), antimicrobial peptides (Audrain et al., 2013), and copper (Yamamoto and Ishihama, 2006). Some of the best characterized members of the Cpx regulon are genes that are involved in envelope protein folding and degradation, further suggesting a role for the Cpx response in maintaining the integrity of the envelope by monitoring and responding to stress due to misfolded envelope proteins. These Cpx-regulated genes include those encoding the periplasmic chaperones CpxP and Spy (Danese and Silhavy, 1998; Raivio et al., 2000), the chaperone/protease DegP/HtrA (Danese et al., 1995), the disulfide bond-forming oxidoreductase DsbA (Pogliano et al., 1997), peptidyl-prolyl isomerase PpiA (Pogliano et al., 1997), IM protease HtpX and YccA, a factor that modulates IM proteolytic activity (Shimohata et al., 2002; Yamamoto and Ishihama, 2006; Raivio et al., 2013). The Cpx response also appears to regulate other ESRs, such as the operon encoding the regulatory components of the σ^E^ response (*rpoE-rseABC*), which is negatively regulated (Price and Raivio, 2009). It is not clear why the σ^E^ response is negatively regulated by the Cpx response, given that both the Cpx and σ^E^ responses respond to stress related to protein misfolding in the envelope. Future studies investigating this cross-regulation may shed more light onto the specific role of these ESRs and the purpose of this regulatory antagonism.

Although ostensibly a “two-component” system, other regulatory factors, such as the periplasmic protein CpxP and the OM lipoprotein NlpE are known to regulate signaling in some capacity in the Cpx response. Overexpression of NlpE is a well-known inducer of the Cpx response in *E. coli* (Danese et al., 1995; Snyder et al., 1995; Danese and Silhavy, 1998). However, NlpE is not essential in signaling all, indeed most, Cpx-inducing cues, such as PapG overexpression (DiGiuseppe and Silhavy, 2003). The crystal structure of NlpE was solved by Hirano et al. (2007) and was shown to possess two distinct β-barrel domains at its N- and C-terminus connected by an unstructured flexible linker. Comparisons of NlpE homologs in Gram-negative organisms reveal that there are two broad types of NlpE: type I (possessing the full-length protein) and type II (lacking the C-terminal domain) (Hirano et al., 2007).

One of the roles that NlpE is thought to play is as a signaler of adhesion to hydrophobic abiotic surfaces, as well as host cell surfaces (Otto and Silhavy, 2002; Shimizu et al., 2016). The structure of NlpE and its implication in sensing surface signals suggest that during signaling, conformational changes in NlpE could lead to the C-terminus “reaching down” and interacting with the sensing domain of CpxA (Hirano et al., 2007). However, the precise mechanism of NlpE signaling to the Cpx response remains largely unknown, although recent papers, that will be discussed shortly, present new insights into the function of NlpE. Interestingly, multi-drug resistant (MDR) strains of *Acinetobacter baumannii* were found to express increased levels of NlpE and this was implicated in higher biofilm formation on abiotic surfaces, despite the fact that the *A. baumannii* NlpE is type II (i.e., lacks a C-terminal domain) (Siroy et al., 2006).

Studies in both the non-pathogenic K-12 strain MC4100 and enterohemorrhagic *E. coli* (EHEC) have suggested NlpE functions as a sensor of surface adhesion both to hydrophobic glass surfaces (K-12, EHEC) and undifferentiated Caco-2 cells (EHEC) (Otto and Silhavy, 2002; Shimizu et al., 2016). Sensing surface adhesion is thought to be an important initial step in the ability of a bacteria to colonize both biotic and abiotic surfaces, particularly as a biofilm (reviewed in Belas, 2014). In line with this, surface sensing by NlpE was implicated by Shimizu et al. (2016) as an important regulatory event in promoting virulence factor expression in EHEC. However, the role of the Cpx response in surface sensing was recently disputed by Kimkes and Heinemann (2018) who did not find induction of Cpx-regulated *yebE-* or *cpxP-*GFP fluorescent reporters in response to adhesion to hydrophobic glass in a microfluidics setup nor when they attempted to recreate the original experimental conditions of Otto and Silhavy (2002). However, a close examination of these studies reveals a number of methodological differences that make it difficult to make a conclusive verdict as to whether or not NlpE is involved in surface sensing. Clearly, these conflicting conclusions point to a need for further study clarifying the role of NlpE in surface adhesion.

Recent studies have pointed to other potential roles for NlpE, specifically, as a sensor for monitoring stress related to lipoprotein trafficking and periplasmic redox state (Grabowicz and Silhavy, 2017; Delhaye et al., 2019). Deleting *lolB*, which encodes for the OM receptor lipoprotein responsible for inserting lipoproteins trafficked to the OM, has a severe deleterious effect on cell growth presumably because essential OM lipoproteins are not trafficked properly (Tanaka et al., 2001; Grabowicz and Silhavy, 2017). Deleting *cpxR* or *nlpE* in genetic backgrounds that suppress this toxicity, restores toxicity (Grabowicz and Silhavy, 2017). Conversely, activating CpxA is able to restore growth in the deleterious *lolB* null background suggesting that the Cpx response, through NlpE, is able to sense lipoprotein trafficking defects and mitigate this stress (Grabowicz and Silhavy, 2017). This makes sense, as NlpE is itself an OM lipoprotein and its own trafficking would be affected by lipoprotein trafficking defects. This model is further supported by work showing that the N-terminal domain of NlpE, which would be physically close to CpxA at the IM in the absence of trafficking to the OM, physically interacts with and is able to activate CpxA (Delhaye et al., 2019). Furthermore, it is known that forcing NlpE to localize to the IM induces the Cpx response (Miyadai et al., 2004; Delhaye et al., 2016). Future studies should work to characterize what Cpx regulon members are responsible for mitigating stress related to lipoprotein mistrafficking. Grabowicz and Silhavy (2017) raised the intriguing possibility that in the absence of the canonical Lol trafficking pathway, an alternative, Cpx-regulated pathway might exist to traffick lipoproteins, as essential OM lipoproteins such as BamD are still trafficked to the OM in the absence of LolB during Cpx-activation.

In addition to this novel role as a sensor for lipoprotein trafficking, NlpE may also act as a sensor for stress related to protein folding. Disulfide bonds in periplasmic proteins are introduced by DsbA, the absence of which leads to Cpx activation (Bardwell et al., 1991; Delhaye et al., 2019). This activation is dependent on the presence of NlpE and NlpE lacking C-terminal cysteine residues (and therefore without its normal disulfide bond) activates the response, suggesting that the function the of the C-terminal domain of NlpE is at least partly as an indicator of periplasmic redox state (Delhaye et al., 2019). It should be noted that the C-terminal cysteine residues of NlpE are conserved across Gram-negative bacteria that possess type I NlpE, suggesting that this function of NlpE may be important in several different organisms (Hirano et al., 2007).

These studies present a model in which NlpE acts as a “Swiss army knife,” with distinct domains involved in sensing distinct stresses and signaling to CpxA to maintain the integrity of the envelope. Although not directly regulating virulence in most cases, these functions of NlpE have potential ramifications on pathogenesis of bacteria at various stages of infection. The transition from the environment to the host may increase oxidative stress or alter periplasmic redox status, necessitating a means to monitor and respond to these changes. A bacterial cell must “know” that it is in contact with an appropriate surface to express virulence factors or form biofilms, making surface sensing a key initial step during infection. Lipoproteins play a key role in the biogenesis of many envelope components, including virulence factors such as secretion machineries, and as such, monitoring lipoprotein trafficking is important for their proper expression. Overall, further studies of the signaling functions of NlpE may reveal important insights into the role that the Cpx response plays as a whole during infection.

CpxP is a periplasmic protein that bears structural homology to the Cpx-regulated periplasmic chaperone Spy (Kwon et al., 2010). The role of CpxP in signaling is thought to be primarily negative. *cpxP* is one of the most highly expressed members of the Cpx regulon upon activation but overexpression of CpxP inhibits activation of CpxA (Raivio et al., 1999, 2013; DiGiuseppe and Silhavy, 2003). This inhibition likely occurs through direct interaction with the periplasmic sensing domain of CpxA (Raivio et al., 1999, 2000; Zhou et al., 2011; Tschauner et al., 2014). It is thought that CpxP, in the presence of misfolded envelope proteins, will be titrated away from CpxA, and subsequently degraded by DegP/HtrA (Buelow and Raivio, 2005; Isaac et al., 2005; Tschauner et al., 2014). However, the role of CpxP in Cpx signaling is, like NlpE, not essential for most studied inducing cues, as inducers such as NlpE overexpression and alkaline pH do not require CpxP to activate the response (DiGiuseppe and Silhavy, 2003). Interestingly, although induction via alkaline pH doesn't require CpxP, *cpxP* mutants are hypersensitized to alkaline pH (Danese and Silhavy, 1998; DiGiuseppe and Silhavy, 2003). Overall, NlpE and CpxP, under the conditions in which they have been studied, rather than acting as integral players in signal transduction, appear to allow for finer regulation of the response, both as a damper of activation, as in the case of CpxP or as an enabler for other sensory inputs, as in the case of NlpE.

Interestingly, recent work has revealed the existence of a conserved, RNase E and Hfq-dependent sRNA encoded in the 3' untranslated region (UTR) of the *cpxP* mRNA, aptly named CpxQ (Chao and Vogel, 2016). In *Salmonella*, CpxQ targeted several envelope proteins, including the sodium-proton antiporter NhaB, the major subunit of the type I pilus FimA, and the periplasmic chaperone protein Skp (Chao and Vogel, 2016). CpxQ was needed for optimal survival in the presence of carbonyl cyanide 3-chlorophenylhydrazone (CCCP), a reagent that disrupts the proton motive force (PMF) at the IM (Chao and Vogel, 2016). This, combined with the regulation of NhaB by CpxQ suggests that its role is connected to preserving the PMF at the IM. In *E. coli*, CpxQ was shown to repress CpxP production by decreasing translation of *cpxP* mRNA (Grabowicz et al., 2016). Furthermore, CpxQ was shown to help combat stress due to a mutant allele of the OM protein LamB [*lamB*(*A23D*)], that aberrantly tethers it to the IM, a lethality that is suppressed by Cpx activation (Cosma et al., 1995; Grabowicz et al., 2016). Interestingly, it was not the regulation of CpxP by CpxQ but repression of the periplasmic chaperone Skp that was responsible for alleviating the toxicity of LamB(A23D) (Grabowicz et al., 2016). Skp is thought to facilitate the aberrant insertion of LamB(A23D) into the IM, creating a pore and disrupting the PMF at the IM, a stress that is relieved by the repression of Skp by CpxQ (Grabowicz et al., 2016). This is consistent with the finding that CpxQ is involved in alleviating stress due to CCCP (Chao and Vogel, 2016).

Overall, these results point to a growing body of evidence for the role of sRNAs in mediating the stress-alleviating effects of ESRs. It had previously been shown that the Cpx response regulates the expression of several sRNAs, such as *cyaR, omrA, omrB*, and *rprA* and that these sRNA are involved in a regulatory network that not only regulates CpxRA but also links it to other 2CST systems such as EnvZ/OmpR (Raivio et al., 2013; Vogt et al., 2014). In the σ^E^ response, σ^E^-regulated sRNAs can directly target and repress porins and OM lipoproteins that are potential sources of envelope stress (Gogol et al., 2011; Guo et al., 2014). Given the demonstrated relevance of sRNAs to envelope stress adaptation, further study of CpxQ presents an opportunity to better understand the mechanism of how the Cpx response alleviates envelope stress.

### The Cpx Response and Bacterial Pathogenesis

#### Escherichia coli

**Figure 5:**
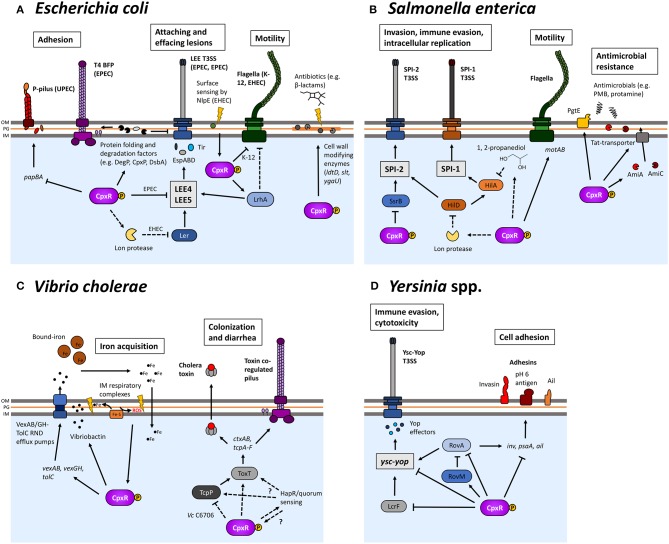
The Cpx response is involved in the pathogenesis of several Gram-negative bacteria. The Cpx response regulates many different processes related to the pathogenesis of several different organisms, such as *Escherichia coli* **(A)**, *Salmonella enterica* **(B)**, *Vibrio cholerae* **(C)**, and *Yersinia* spp. **(D)**. Cpx regulation not only regulates the assembly of envelope-localized virulence determinants but also several other processes that impact envelope integrity and affect the ability of bacteria to colonize hosts. Proposed positive regulation is indicated by regular arrows, whereas blunt arrows represent negative regulation. Dashed lines indicate where regulation is indirect.

The relevance of the Cpx response to the physiology and pathogenesis of various strains of *Escherichia coli* is well-documented (Figure 5A). Studies in MP1, a commensal strain of *E. coli* isolated from mice, found that deleting *cpxR* caused a severe colonization defect in mice (Lasaro et al., 2014). Further, *cpxRA* deleted strains of UPEC are attenuated in colonization and virulence in both mouse and zebrafish models (Debnath et al., 2013).

At the molecular level, multiple studies have focused on envelope-localized protein machineries such as the UPEC P-pilus and the enteropathogenic *E. coli* (EPEC) type IV bundle-forming pilus (BFP), which are assembled at the envelope and play key roles in adhesion to host cells (Wullt et al., 2000; Tobe and Sasakawa, 2001). Overexpressing PapE and PapG subunits of the P pilus activates the Cpx response but the activation of the Cpx response is not due to non-specific protein misfolding stress but rather the presence of a specific N-terminal extension on PapE involved in pilus subunit polymerization, suggesting that the Cpx response specifically monitors pilus assembly (Jones et al., 1997; Lee et al., 2004). In support of this, in the absence of *cpxR*, K-12 strains containing P pili genes produce shorter P pili, suggesting that the Cpx response is needed for efficient pilus expression and assembly (Hung et al., 2001). CpxR is also able to repress the expression of the *pap* pilin genes directly (Hernday et al., 2004). Similar roles for the Cpx response in both efficient assembly and inhibition of the elaboration of pili have been demonstrated in relation to the BFP of EPEC, despite their differing assembly and structural composition compared to P-pili (Nevesinjac and Raivio, 2005; Humphries et al., 2010; Vogt et al., 2010). In particular, efficient expression of the BFP was dependent on the Cpx-regulated periplasmic protein folding factors CpxP, DsbA, and DegP/HtrA (Vogt et al., 2010). The regulation of multi-protein envelope complexes by the Cpx response is not limited to pili, as flagella in several strains of *E. coli* are shown to be negatively regulated (De Wulf et al., 1999; Price and Raivio, 2009; Shimizu et al., 2016) and the Cpx response also regulates secretion machinery in several pathogenic *E. coli* (see below).

EPEC and EHEC are known to cause attaching and effacing (A/E) lesions on host intestinal tissue that are dependent on the locus of enterocyte effacement (LEE) T3SS (McDaniel et al., 1995; McDaniel and Kaper, 1997). Activation of the Cpx response represses LEE T3S in EPEC (Macritchie et al., 2008). This regulation occurs through the repression of the transcription of several LEE operons and post-transcriptionally by the action of the periplasmic chaperone and protease DegP/HtrA (Macritchie et al., 2008; MacRitchie et al., 2012). The regulation of the LEE T3SS does have common features between EPEC and EHEC, namely the transcriptional regulator Ler (Mellies et al., 1999; Abe et al., 2008). However, recent studies in enterohaemorrhagic *E. coli* (EHEC) have led to contrasting conclusions as to the involvement of the Cpx response in regulating the LEE (De la Cruz et al., 2016; Shimizu et al., 2016). De la Cruz et al. (2016) found that increased levels of phosphorylated CpxR in the absence of CpxA repressed levels of LEE5-encoded EspABD translocator proteins as well as transcription of *ler*, in a manner dependent on the protease Lon. Overexpressing NlpE lead to a reduction in *espA* transcript as quantified by qPCR. However, Shimizu et al. (2016) found that *espA-luxE* reporter activity was activated in response to adhesion to hydrophobic glass beads and undifferentiated Caco-2 cells in a manner dependent on NlpE and CpxA. In adhered cells, NlpE activated *espA* transcription (Shimizu et al., 2016). This led to the suggestion that activating the Cpx response after surface sensing by NlpE leads to an upregulation of LEE T3SS genes, specifically by CpxR regulation of the positive regulator LhrA (Shimizu et al., 2016). It is possible that the difference between these two studies is due to the fact that in most experiments, surface-adhered EHEC cells were used in Shizimu and colleagues' study, whereas the study of De la Cruz et al. was conducted mostly on cells grown in liquid media. More work should be conducted to elucidate the impact that surface sensing has on modulating Cpx signaling, as it appears that adhesion significantly alters the Cpx regulation of virulence in EHEC.

A growing body of evidence highlights the relevance of the Cpx response to pathogenesis beyond protein quality control in the envelope. Studies of the Cpx regulon in *E. coli* have shown that several genes involved in cell wall modifications are upregulated by Cpx activation (Raivio et al., 2013; Bernal-Cabas et al., 2015). These include D-alanyl-D-alanine carboxypeptidase DacC (penicillin binding protein (PBP) 6) (Pedersen et al., 1998), L,D-transpeptidase LdtD (Magnet et al., 2008), lytic transglycosylase Slt (Höltje et al., 1975) and YgaU, a hypothetical protein with a LysM domain predicted to be involved in cell wall degradation (Buist et al., 2008). Accordingly, the Cpx response is activated when *ygaU* and/or *ldtD* are deleted and in strains that lack PBPs 4, 5 7 and AmpH (Evans et al., 2013; Bernal-Cabas et al., 2015). Activating the Cpx response increased diaminopimelic acid (DAP)-DAP crosslinks formed by LdtD, suggesting that Cpx activation has direct ramifications on the structural composition of PG (Bernal-Cabas et al., 2015).

It was recently shown that deleting *cpxR* increased susceptibility to the β-lactam antibiotic mecillinam, but constitutive activation led to defects in cell shape, growth, and division (Delhaye et al., 2016). Moreover, these defects were dependent on the expression of LdtD (Delhaye et al., 2016). These results point to a role for the Cpx response in monitoring not only protein quality control in the envelope, but also as a key regulatory factor for proper cell wall integrity. It has been observed that the Cpx response regulates amidases in *P. aeruginosa* and *S*. Typhimurium, suggesting that Cpx regulation of cell wall homeostasis may be a conserved function across Gram-negative organisms (Weatherspoon-Griffin et al., 2011; Yakhnina et al., 2015). The cell wall is essential for maintaining cell shape and preventing lysis during shifting osmotic conditions and is an important target for many antibiotics and innate immune strategies. Upregulation of PG-modifying enzymes may serve to reinforce the envelope during stress in other compartments of the envelope so as to increase its overall stability. Interestingly, it was recently shown that increased levels of DAP-DAP crosslinkages increases resistance to lysozyme (Stankeviciute et al., 2019). It is possible that Cpx-regulation of *ldtD* and *ygaU* may be a strategy for dealing with envelope stress arising from host innate immunity. Although the full picture remains to be seen, these studies point to the Cpx response as a fine-tuning regulator of PG structure that serves to mitigate potential stresses to the cell wall, both in and out of hosts.

#### Citrobacter rodentium

The presence of shared virulence determinants, most notably the LEE, in the murine pathogen *Citrobacter rodentium*, has led to its use as a model to study EPEC and EHEC infections *in vivo* (reviewed in Collins et al., 2014). Accordingly, the *in vivo* contribution of the Cpx response to virulence is arguably best demonstrated by recent studies in *C. rodentium*. The role of the Cpx response in promoting *in vivo* fitness and virulence has also previously been demonstrated in other organisms, such as in uropathogenic *E. coli* (Debnath et al., 2013), *Salmonella* (Fujimoto et al., 2018), and gonococcal *Neisseria*, where MisRS, a 2CST system suggested to be the *Neisseria* CpxRA homolog, was needed to establish infection in the genital tract of mice (Kandler et al., 2016; Gangaiah et al., 2017).

Deleting *cpxRA* attenuates the ability of *C. rodentium* to colonize and cause death in mice, in some cases completely (Thomassin et al., 2015, 2017; Vogt et al., 2019). *cpx* gene expression is induced during infection suggesting that the response is activated during host colonization (Thomassin et al., 2015, 2017). Deleting *cpxP* or *nlpE* had no significant impact on colonization, virulence or ability to bind to HeLa cells unlike the *cpxRA* null mutant which was attenuated in all these aspects (Giannakopoulou et al., 2018). Attenuation of virulence was found to be independent of type III secretion and growth rate, as secretion profiles and growth rates remain relatively the same as compared to wildtype in *cpxRA* mutants (Thomassin et al., 2015; Vogt et al., 2019).

Interestingly, it was recently reported that deleting the Cpx-regulated genes *degP/htrA* and *dsbA* led to a reduction in secreted protein levels of the T3SS translocator protein EspB in *C. rodentium* (Vogt et al., 2019). Furthermore, deleting *degP* or *dsbA* caused *C. rodentium* to become completely avirulent, unlike *cpxRA*, which in this study resulted in less attenuation of virulence compared to previous studies (Thomassin et al., 2015, 2017; Vogt et al., 2019). The reason for the difference between these studies is currently unknown. However, mutating the promoter of *degP* and *dsbA* such that they are no longer under the control of CpxR does not attenuate virulence, suggesting that Cpx regulation of *degP* or *dsbA* is not the entire reason why these mutants are avirulent (Vogt et al., 2019). Overall, these studies point to a key role for the Cpx response in promoting *in vivo* fitness and virulence, but further work characterizing the mechanism of how this occurs is needed.

#### Salmonella

The Cpx response has been strongly implicated in the regulation of virulence in *Salmonella enterica*, which include *S. enterica* serovar Typhi and Typhimurium (*S*. Typhi and *S*. Typhimurium, respectively; Figure 5B). In both *S*. Typhi and Typhimurium, virulence is heavily dependent on two pathogenicity islands SPI-1 and SPI-2, both of which encode T3SS that facilitate invasion, immune invasion and intracellular survival (reviewed in McGhie et al., 2009; Que et al., 2013; Jennings et al., 2017). *cpx* genes in *Salmonella* are organized as they are in *E. coli* (Nakayama et al., 2003). The sequences of *cpxRA* and *cpxP* have 97, 96, and 88% identity, respectively, to their corresponding homologs in *E. coli* (Nakayama et al., 2003). As in *E. coli*, overexpression of the OM lipoprotein NlpE activates the Cpx response (Humphreys et al., 2004). CacA (Cpx-activating connector-like factor A), a small protein conserved in several Gram-negative species including *E. coli*, is induced by RpoS and is needed for full expression of Cpx-dependent genes such as *cpxP* and *spy* in *Salmonella*, suggesting that fine-tuning of Cpx regulation occurs by input from other regulatory systems, for example, in response to growth phase (Kato et al., 2012). Similarly, in *E. coli*, RpoS positively regulates *cpxRA*, further suggesting that Cpx regulation occurs in conjunction with other regulatory systems (De Wulf et al., 1999).

Transposon insertions in *cpxA* were identified in a screen of mutants of *S*. Typhi with lowered ability to invade INT407 cells (LeClerc et al., 1998). In *S*. Typhimurium, deletion of *cpxA* but not *cpxR* at low pH (pH 6.0) strongly represses the expression of a *hilA-lacZ* reporter, leading to a loss of invasion-effector protein expression and a severe defect in ability to invade INT407 cells. HilA is a key activator for SPI-1, which encodes T3SS responsible for secreting SipBCD translocator proteins and facilitating invasion (Que et al., 2013). These observations prompted the suggestion that CpxA repression of SPI-1 was CpxR-independent (Nakayama et al., 2003). However, several recent lines of evidence show that the regulation of SPI-1 by CpxA is not independent of CpxR. Deleting *cpxRA* mimics the phenotype of *cpxR* not *cpxA* mutants and overexpressing NlpE represses the expression of SPI-1 (De la Cruz et al., 2015; Subramaniam et al., 2019). Further, deleting the AckA-Pta (acetate kinase, phosphotransacetylase) pathway responsible for generating acetyl phosphate mitigates the effect of deleting *cpxA*, suggesting that in the absence of CpxA phosphatase activity, non-specific phosphodonors activate CpxR, a phenomenon previously noted (McCleary and Stock, 1994; Danese and Silhavy, 1998; Wolfe et al., 2008; De la Cruz et al., 2015). The repression of SPI-1 by phosphorylated CpxR was dependent on Lon, a protease that degrades HilD, a positive regulator of HilA, suggesting that Cpx regulation of SPI-1 occurs by regulating the stability of HilA (De la Cruz et al., 2015). Recently, a study of the Cpx regulon in *S*. Typhimurium suggested that *pocR*, a negative regulator of the *pdu-cob* cluster that encodes for genes involved in the breakdown of 1,2-propanediol (PDO), is CpxR-regulated (Subramaniam et al., 2019). PDO has been shown to repress *hilA* (Nakayama and Watanabe, 2006), suggesting that Cpx regulation of SPI-1 may occur with inputs from metabolism, although the precise mechanism of how this occurs is not clear.

Activation of the Cpx response was also shown to repress SsrB, a key activator for SPI-2 found on SPI-2 itself (Yoon et al., 2009; De la Cruz et al., 2015). It appears that this repression of SPI-2 may occur through two mechanisms, by CpxR directly binding the promoter of *ssrB* (Subramaniam et al., 2019) and by the repression of HilD, which has been shown to activate both SPI-1 and SPI-2 (Bustamante et al., 2008; De la Cruz et al., 2015). Interestingly, CpxR positively regulates motility in *Salmonella*, a finding that stands in contrast to observations in *E. coli* (Subramaniam et al., 2019).

Antimicrobial peptides (AMPs) are among the many challenges enteric pathogens face as they transition to the intestinal environment. These AMPs cause stress primarily by disrupting the envelope, and in particular, disrupt important processes such as ATP generation (reviewed in Li et al., 2017). It makes sense, then, that the Cpx response is important for mediating resistance to various antimicrobial compounds. The Cpx response is activated by polymyxin B (Fujimoto et al., 2018). CpxR was found to regulate *pgtE*, an OM protease implicated in cleaving and inactivating antimicrobial peptides and deleting *cpxR* accordingly increased sensitivity to polymyxin B (Subramaniam et al., 2019). NlpE overexpression increased resistance to antimicrobial peptides protamine, magainin 2 and melittin by upregulating two twin-arginine transport (Tat)-dependent N-acetyl muramoyl-L-alanine amidases AmiA and AmiC in a CpxR-dependent manner (Weatherspoon-Griffin et al., 2011). As these AMPs are an important component of innate immunity, overcoming this barrier is key for pathogens attempting to establish an infection. It appears that the Cpx response may play an important role in overcoming this challenge, a suggestion supported by other studies that have implicated the Cpx response in mediating resistance to AMPs, such as studies of MisRS, a CpxRA homolog in *Neisseria* spp. (Kandler et al., 2016).

It was initially reported that deleting *cpxR* did not affect colonization of mice organs relative to WT (Humphreys et al., 2004). Interestingly, a recent *in vivo* study in a streptomycin-treated mouse model found that while CpxRA was not needed to cause colitis, it was needed for colonization (Fujimoto et al., 2018). It is likely that the differences between these studies are due to the mouse model used in their experiments. While streptomycin-treated mice infected with *S*. Typhimurium cause symptoms analogous to the gastroenteritis caused by *S*. Typhimurium in humans, non-cleared mice infected with *S*. Typhimurium display a typhoid-like illness (Barthel et al., 2003). Further *in vivo* work characterizing the contribution of the Cpx response to *Salmonella* virulence is needed to elucidate the causes of the differences between these models of infection.

#### Vibrio cholerae

*Vibrio cholerae* is a Gram-negative enteric pathogen and the causative agent of the diarrheal disease cholera (reviewed in Clemens et al., 2017). In recent years, a number of studies have examined the impact of the Cpx response in *V. cholerae*. The basic genomic organization of *cpx* genes resembles that of *E. coli* and *S. enterica*: *cpxRA* is encoded as an operon with *cpxP* encoded upstream and transcribed divergently (Slamti and Waldor, 2009). The sequences of *cpxA, cpxR* and *cpxP* possess 43.6, 60.3, and 21.6% identity, respectively, to their *E. coli* counterparts (Slamti and Waldor, 2009). Importantly, the degree of conservation in the predicted periplasmic sensing domain of *cpxA* is far lower (20.7%) compared to the cytoplasmic region responsible for CpxR phosphorylation (54.3%), suggesting potential differences in CpxA-activating signals (Slamti and Waldor, 2009). Unlike in *E. coli*, NlpE in *V. cholerae* is type II (lacking a C-terminal domain) (Hirano et al., 2007) and NlpE overexpression does not activate the Cpx response in *V. cholerae* (Slamti and Waldor, 2009). Similarly, alkaline pH is also not a Cpx pathway inducer in *V. cholerae* (Acosta et al., 2015b). The Cpx response in *V. cholerae* is activated in response to CuSO_4_, chloride ions, aberrant disulfide bond formation, iron chelation, the absence of RND (resistance-nodulation-division) efflux pumps and ROS (Slamti and Waldor, 2009; Taylor et al., 2014; Acosta et al., 2015b; Kunkle et al., 2017). Mutations in DsbD, which is involved in the folding of secreted proteins by mediating disulfide bond formation (Ito and Inaba, 2008) and TolC, the OM component of efflux pumps (Koronakis et al., 2004), were enriched in a screen of mutants that activated the Cpx response, suggesting a conserved role for Cpx regulation of envelope homeostasis in *V. cholerae* (Slamti and Waldor, 2009).

The Cpx response has been implicated in regulating several processes that are important for the survival and growth of *V. cholerae* as it infects its host (Figure 5C). An important innate immune strategy is the sequestration of important minerals to limit bacterial growth. Iron, in particular, is key to several metabolic processes and is normally limited in hosts as it is sequestered in heme groups or iron-carrying proteins such as ferritin. As such, iron uptake presents an important challenge for enteric pathogens to overcome to establish infection (reviewed in Hood and Skaar, 2012). The Cpx response has been implicated in adapting to the stresses caused by low iron. The Cpx response in *V. cholerae* is activated in response to the chelation of iron and the Cpx regulon in *V. cholerae* O1 El Tor C6706 is enriched in genes involved in iron acquisition and metabolism, such as those involved in biosynthesis of the siderophore vibriobactin, ferrichrome transport and heme uptake (Acosta et al., 2015b). Furthermore, supplementing growth media with an excess of FeSO_4_ was able to decrease activation of the response not only in iron-limiting conditions by 2,2'-bipyridyl, but also in response to diamide and RND efflux pump deletions suggesting that these Cpx-activating cues are related in some way to iron uptake (Acosta et al., 2015b).

*V. cholerae* possess six RND efflux systems, VexAB, CD, EF, GH, IJK, and LM, all of which likely utilize the OM protein TolC as their OM pore (Bina et al., 2008). These efflux pumps are responsible for the efflux of a variety of potentially harmful substances, including antibiotics such as polymyxin B, erythromycin and ampicillin and detergents such Triton X-100 and sodium dodecyl sulfate (SDS) (Bina et al., 2008; Taylor et al., 2012). Loss of these RND efflux pumps leads to a reduction in cholera toxin (CT) and toxin coregulated pilus (TCP) production and abolishes *V. cholerae*'s ability to colonize in an infant mouse model (Bina et al., 2008). Deletions of *tolC* and genes encoding RND efflux pumps VexAB and VexGH activate the Cpx response and likewise, activating the Cpx response induces expression of TolC and VexAB and VexGH (Slamti and Waldor, 2009; Taylor et al., 2014; Acosta et al., 2015b). These results strongly link the Cpx response to *V. cholerae*'s efflux machinery. However, although inducing the Cpx response by KCl or by a cpxA^*^ mutation was able to increase growth on thiosulfate-citrate-bile sucrose (TCBS) agar, a medium which requires the action of RND efflux to allow for growth, deleting *cpxR* did not negatively affect growth on TCBS agar relative to WT suggesting that the Cpx response is not normally required for growth in efflux-requiring conditions (Taylor et al., 2014). Thus, although efflux and the Cpx response are strongly linked genetically, the extent to which the Cpx response is linked to innate antimicrobial resistance in *V. cholerae* remains to be determined.

Interestingly, the significance of Cpx regulation of RND efflux pumps was shown to be connected to iron acquisition and transport (Kunkle et al., 2017). Mutations in genes involved in the synthesis of vibriobactin, a catechol siderophore (Griffiths et al., 1984), were found to suppress Cpx activation in efflux-deficient mutants (Kunkle et al., 2017). Levels of extracellular vibriobactin were reduced in RND efflux negative mutants, suggesting RND efflux pumps function to transport vibriobactin out of the cell and that in their absence, vibriobactin aberrantly accumulates in the periplasmic space (Kunkle et al., 2017). As iron is an essential component of several IM complexes of the electron transport chain (ETC) (Friedrich et al., 2016), it was suggested that the activation of the Cpx response in the RND-deficient mutant was due to the chelation of iron away from these complexes, leading to aberrant protein folding at the IM and/or the production of ROS during respiration (Kunkle et al., 2017). Consonant with this hypothesis is the observation that activation in efflux-negative mutants was abolished in anaerobic growth and in a *sdhA* mutant, which encodes for a subunit of succinate dehydrogenase (Kunkle et al., 2017). Studies in EPEC have shown that respiratory complexes found at the IM, such as NDH-I and cytochrome *bo*_3_ are negatively regulated by the Cpx response, suggesting that monitoring respiratory complexes at the IM may be a conserved function of the Cpx response, although it remains to be seen how the Cpx response senses these stresses (Raivio et al., 2013; Guest et al., 2017).

Elucidating the precise role of the Cpx response in regulating virulence in *V. cholerae* is complicated by contrasting results in different, albeit closely related strains. The primary diarrheagenic effect of *V. cholerae* stems from the ADP-ribosylating action of CT, which leads to changes in intracellular signaling and fluid secretion from intestinal epithelial cells (Field et al., 1972; and Gill et al., 1978). RND efflux pumps have been shown to be needed for the optimal production of CT and TCP in O1 El Tor strain N16961, but deleting *cpxR* in a strain lacking all six RND efflux pumps did not affect decreased levels of CT and TCP, suggesting a Cpx-independent mechanism for the regulation of CT and TCP production (Bina et al., 2008; Taylor et al., 2014). Furthermore, neither cpxA^*^ (constitutively active) or *cpxR* mutants changed CT or TCP levels relative to WT (Taylor et al., 2014). Accordingly, neither deletions of *cpxR, cpxA* or *cpxP* or activation of the response via a cpxA^*^ allele affected the ability of N16961 to colonize infant mice (Slamti and Waldor, 2009).

In contrast to these results, mutating *cpxR* in a related O1 El Tor strain, C6706, led to an increase in *ctxB, tcpA*, and *tcpP* transcription (Acosta et al., 2015a). Correspondingly, overexpressing CpxR led to a complete abrogation of the expression of CT and TCP both at the transcriptional and protein level (Acosta et al., 2015a). CpxR overexpression repressed the transcription of ToxT, the direct regulator of TCP and CT (DiRita et al., 1991) and TcpP, a regulator involved in promoting the expression of ToxT (Häse and Mekalanos, 1998), suggesting that Cpx regulation of virulence occurs through established virulence regulators (Acosta et al., 2015a). It was suggested that the discrepancy in results between C6706 and N16961 could be due to a known defect in the ability of N16961 to quorum sense, another process closely involved in regulating virulence in *V. cholerae* (Zhu et al., 2002). Nonetheless, these contrasting results underscore the need to be cognizant of strain differences when conducting work on even closely related strains and future studies investigating these differences may help elucidate the precise mechanisms of Cpx regulation of key *V. cholerae* virulence determinants.

#### *Yersinia* spp.

The genus *Yersinia* include a number of Gram-negative pathogens including *Y. pestis, Y. pseudotuberculosis* and *Y. enterolitica*. Studies of the Cpx response have focused on the latter two species, *Y. pseudotuberculosis* and *Y. enterolitica*, which, while not as well-known as the infamous cause of the bubonic and pneumonic plagues, are nonetheless significant pathogens of humans (Drummond et al., 2012). However, a study in *Y. pestis* has implicated the Cpx response in promoting survival in neutrophils (O'Loughlin et al., 2010), and as such it is certainly possible that the findings in other species of *Yersinia* are applicable to *Y. pestis*.

The virulence of *Yersinia* spp. depends heavily on the action of the virulence plasmid-encoded Ysc-Yop (*Yersinia* secretion-*Yersinia* outer protein) T3SS. Ycs proteins form the machinery responsible for the translocation of a collection of different Yop effectors that block phagocytosis into host cells directly and allow for extracellular replication of *Yersinia* (reviewed in Cornelis, 2002). *Yersinia* spp. are thought to primarily be extracellular pathogens, although they possess the ability to invade and survive intracellularly (Grabenstein et al., 2004). Several ESRs contribute to virulence factor regulation in *Yersinia*, including the Psp and Rcs responses (Flores-Kim and Darwin, 2012; Li et al., 2015b).

In the last two decades, several studies have examined the Cpx response in *Y. enterolitica* and *Y. pseudotuberculosis*, particularly in relation to type III secretion and adhesion to host cells (Figure 5D). The sequence identity of *cpxR* and *cpxA* to their *E. coli* homologs are high in both *Y. pseudotuberculosis* and *Y. enterolitica* (89 and 80%, respectively in *Y. pseudotuberculosis* and 81 and 74%, respectively in *Y. enterolitica*) (Heusipp et al., 2004; Carlsson et al., 2007a). As in *E. coli*, the Cpx regulon in *Yersinia* spp. includes envelope protein folding factors and proteases such as *htrA*/*degP, dsbA* and *ppiA* (Heusipp et al., 2004; Carlsson et al., 2007a,b; Liu et al., 2011).

Several studies have shown that deleting *cpxA* represses the production of the Ysc secretion machinery, Yop effectors as well as adhesins such as invasin (*inv*) in *Y. pseudotuberculosis* (Carlsson et al., 2007a,b; Liu et al., 2011, 2012). Furthermore, deleting *cpxA* abrogates the ability of *Y. pseudotuberculosis* to cause cytotoxicity in HeLa cells caused by YopE, a type III secretion secreted cytotoxin (Carlsson et al., 2007a,b; Vlahou et al., 2009; Liu et al., 2012). This repression of virulence occurs in the *cpxA* mutant as a result of non-specific phosphorylation of CpxR in the absence of CpxA phosphatase activity, as constitutively active cpxA^*^ mutants produce similar results to *cpxA* knock-out mutants, and removing acetyl-phosphate by introducing a Δ*ackA, pta* mutation abolishes the effects of deleting *cpxA* (Carlsson et al., 2007a,b; Liu et al., 2011, 2012). Cytotoxicity was not affected in a *cpxR* null mutant and this mutation actually increased cell adhesion and invasion (Carlsson et al., 2007a,b). Furthermore, ectopically expressing CpxR, but not CpxR D51A (unable to be phosphorylated), repressed cytotoxicity, indicating that phosphorylated CpxR is responsible for the repression of cell adhesion and type III secretion (Carlsson et al., 2007a; Liu et al., 2011). These results suggest that the Cpx response functions to repress T3SS assembly and secretion and are corroborated by transcriptional data which shows that *cpxR* is downregulated during conditions that induce the expression of the Ysc-Yops T3SS (Carlsson et al., 2007a).

The precise mechanism of regulation of these processes appears to require a multitude of different regulatory approaches. Although Ysc structural protein levels are reduced in membrane fractions in *cpxA* null mutants, the promoters of these genes lack CpxR binding sites, suggesting indirect regulation at the transcriptional level or potential post-transcriptional regulation by other Cpx-induced factors (Carlsson et al., 2007a; Liu et al., 2012). In contrast, the transcription of several *yop* effector genes and *syc* effector chaperones are directly regulated by phosphorylated CpxR (Liu et al., 2012). In addition, LcrF, a transcription factor which induces the expression of *yop* genes when the temperature shifts from 26 to 37°C (Cornelis et al., 1989), is also directly regulated by CpxR suggesting that regulation of Yop effectors occurs not only through directly modifying transcription but also by modulating expression of other transcriptional activators (Liu et al., 2012).

A similar story can be told in regards to cell adhesion. Phosphorylated CpxR can directly modulate transcription of known adhesins invasin and pH 6 antigen (*psaA*), as well as proposed adhesins such as the OM protein Ail, suggesting direct regulation (Carlsson et al., 2007b; Liu et al., 2011). However, regulation of these factors also occurs through other regulators. RovA, a global transcriptional regulator that activates the expression of invasin, is directly regulated by CpxR (Carlsson et al., 2007b). A further level of control was recently discovered when it was demonstrated that RovM, a regulator of RovA that represses its expression during nutrient limitation, was found to be directly CpxR-P regulated (Heroven and Dersch, 2006; Heroven et al., 2008; Thanikkal et al., 2019). Thus, RovA expression is not only modulated by CpxR at its own promoter, but also through CpxR upregulation of RovM. The *in vivo* significance of this result may be related to the finding that the RovM regulatory cascade is implicated in causing a lifestyle switch from acute to persistent infection in mice (Avican et al., 2015). This is also consistent with findings in *S*. Typhimurium, where the Cpx response was dispensable for causing acute virulence in mice, but was needed to colonize the mouse gut over longer spans of time (Fujimoto et al., 2018). The sum of these results points to a network of regulatory connections that control the expression of several key *Yersinia* virulence determinants. An area for further investigation is elucidating the precise inducing cues of the Cpx response in *Yersinia* spp. so as to understand why the Cpx response negatively regulates key envelope localized virulence determinants.

The Cpx response in *Y. enterolitica* has not been explored as thoroughly as in its sister species. Nonetheless, studies point to some key differences. Unlike in *Y. pseudotuberculosis*, to our knowledge, no studies have been published successfully deleting *cpxA* in *Y. enterolitica*, and attempts to do so even in the presence of CpxA expression in *trans* have not been successful (Ronnebaumer et al., 2009). Furthermore, overexpression of CpxA is deleterious even in normal growth conditions, suggesting that the effect of deleting and overexpressing CpxA is related to the phosphorylation of CpxR (Ronnebaumer et al., 2009). In *Y. pseudotuberculosis*, however, deleting *cpxA* only causes a minor growth defect (Carlsson et al., 2007a). At this point, it is unclear what is responsible for the apparent differences between *Y. pseudotuberculosis* and *Y. enterolitica*. However, as the Cpx regulon remains relatively uncharacterized in both of these species, its further characterization may yield insights into the root of these differences.

#### Summary

It should be noted that studies of the Cpx response are not limited to the organisms we discuss in this review. Unfortunately, given restraints on space, it is impossible to discuss all of the relevant work in other organisms. As such, we wish to point readers to the following studies for further reading: *Shigella* spp. (Nakayama and Watanabe, 1995, 1998; Mitobe et al., 2005), *Legionella pneumophila* (Gal-Mor and Segal, 2003; Vincent et al., 2006; Altman and Segal, 2008)*, Haemophilus ducreyi* (Labandeira-Rey et al., 2009, 2010; Gangaiah et al., 2013), and *Neisseria* spp. (Tzeng et al., 2004, 2008; Kandler et al., 2016; Gangaiah et al., 2017).

The work conducted in a diverse array of different organisms highlights the contribution of the Cpx response to the virulence of several Gram-negative pathogens. Although the exact details can differ between organisms, or even closely related strains, there are a few important motifs that we see repeated throughout these studies. The Cpx regulon across species typically includes envelope-localized protein folding and degradation factors, such as DegP/HtrA. There exists a consistent link between envelope-localized, multi-protein complexes, such as pili or secretion machinery, and regulation by the Cpx response. These patterns support an overarching paradigm where the Cpx response functions to maintain envelope integrity by monitoring protein quality control. However, the specific effect of the Cpx response in different organisms is not uniform, despite the fact that essentially all of the organisms surveyed in this review are enteric pathogens that, generally speaking, share a similar niche in the host. In some organisms (such as *C. rodentium*), the Cpx response is indispensable to colonization and virulence *in vivo*, while in other organisms, activation of the Cpx response has a predominantly negative impact on key virulence determinants. Related to this, while in general, the Cpx response negatively regulates key virulence determinants, the presence of the Cpx response tends to be positively associated with colonization or long-term persistence. Given this general observation, it appears that the role of the Cpx response in pathogenesis tends not toward promoting virulence factor expression, but rather ensuring that the cell can survive in the midst of the multitude of stresses faced during the course of infection, whether that be due to the expression of the virulence factors themselves or from external factors, such as host immune strategies.

What is responsible for the observed differences between organisms? It is almost certain that phylogenetic differences can explain aspects of the observed diversity. For example, remarkable similarities exist in the mechanism of Cpx regulation of the LEE T3SS in pathogenic *E. coli* and the SPI-1 T3SS in *S*. Typhimurium (see Figures 4, 5 and the corresponding sections). But when comparing more distantly related species (as is reflected in greater sequence dissimilarity in *cpx* genes), such as *E. coli* and *V. cholerae*, more disparities tend to be seen in inducing cues, mechanisms of signaling or regulon members. It is also highly likely that differences in life-cycle and infection strategies influence how the Cpx response functions in different species. For example, while phylogenetically, *E. coli* may be more closely related to *Salmonella*, than to *V. cholerae*, the extracellular lifestyle adopted by EPEC and EHEC is more analogous to *V. cholerae* than to the invasive lifestyle of *Salmonella*. And so, commonalities, such as the regulation of pilus expression, can be seen in *E. coli* and *V. cholerae*. Perhaps part of the issue is that the Cpx response is best characterized in various strains of *E. coli* and studies in other pathogens have yet to be conducted to the same level of depth. If this is the case, further study in these organisms will only yield expanded insight into our understanding of the Cpx response and its function in the physiology and pathogenesis of a variety of different organisms.

## Concluding Remarks

It is clear from the literature that ESRs such as the σ^E^ response and the Cpx response play key roles in the physiology and pathogenesis of several Gram-negative bacteria. Nonetheless, it is also clear that many questions remain about these ESRs, such as those about precise mechanisms of signaling and regulation of target genes and processes, and their relevance to *in vivo* fitness. Particularly of interest is exploring the precise points at which these ESRs are relevant to colonization and infection. When and where are these ESRs active? What precise cues *in vivo* induce these responses? Do ESRs predominantly mitigate encountered stresses or do ESRs play more proactive roles in promoting colonization or virulence? Exploring these questions further will not only increase our basic understanding of these Gram-negative bacteria but also may help provide potential strategies for the prevention and treatment of the diseases caused by these organisms.

## Author Contributions

CH and TC reviewed the literature, co-wrote the manuscript, and prepared the figures. GR and TR conceived the idea and co-edited the drafts.

### Conflict of Interest Statement

The authors declare that the research was conducted in the absence of any commercial or financial relationships that could be construed as a potential conflict of interest.
